# Clinical-Grade Peptide-Based Inhibition of CK2 Blocks Viability and Proliferation of T-ALL Cells and Counteracts IL-7 Stimulation and Stromal Support

**DOI:** 10.3390/cancers12061377

**Published:** 2020-05-27

**Authors:** Yasser Perera, Alice Melão, Ailyn C. Ramón, Dania Vázquez, Daniel Ribeiro, Silvio E. Perea, João T. Barata

**Affiliations:** 1 Laboratory of Molecular Oncology, Center for Genetic Engineering and Biotechnology, Havana 10600, Cuba; yasser.perera@cigb.edu.cu (Y.P.); ailyn.ramon@cigb.edu.cu (A.C.R.); silvio.perea@cigb.edu.cu (S.E.P.); 2 Instituto de Medicina Molecular João Lobo Antunes, Faculdade de Medicina, Universidade de Lisboa, 1649-028 Lisbon, Portugal; alice.melao@gmail.com (A.M.); danielribeiro@medicina.ulisboa.pt (D.R.); 3 Pharmacogenomics Department, Center for Genetic Engineering and Biotechnology, Havana 10600, Cuba; dania.vazquez@cigb.edu.cu

**Keywords:** T-cell acute lymphoblastic leukemia (T-ALL), CIGB-300, Casein kinase 2 (CK2), IL-7 receptor (IL-7R), IL-7-mediated signaling, Stromal support, Signaling therapies

## Abstract

Despite remarkable advances in the treatment of T-cell acute lymphoblastic leukemia (T-ALL), relapsed cases are still a major challenge. Moreover, even successful cases often face long-term treatment-associated toxicities. Targeted therapeutics may overcome these limitations. We have previously demonstrated that casein kinase 2 (CK2)-mediated phosphatase and tensin homologue (PTEN) posttranslational inactivation, and consequent phosphatidylinositol 3-kinase (PI3K)/Akt signaling hyperactivation, leads to increased T-ALL cell survival and proliferation. We also revealed the existence of a crosstalk between CK2 activity and the signaling mediated by interleukin 7 (IL-7), a critical leukemia-supportive cytokine. Here, we evaluated the impact of CIGB-300, a the clinical-grade peptide-based CK2 inhibitor CIGB-300 on T-ALL biology. We demonstrate that CIGB-300 decreases the viability and proliferation of T-ALL cell lines and diagnostic patient samples. Moreover, CIGB-300 overcomes IL-7-mediated T-ALL cell growth and viability, while preventing the positive effects of OP9-delta-like 1 (DL1) stromal support on leukemia cells. Signaling and pull-down experiments indicate that the CK2 substrate nucleophosmin 1 (B23/NPM1) and CK2 itself are the molecular targets for CIGB-300 in T-ALL cells. However, B23/NPM1 silencing only partially recapitulates the anti-leukemia effects of the peptide, suggesting that CIGB-300-mediated direct binding to CK2, and consequent CK2 inactivation, is the mechanism by which CIGB-300 downregulates PTEN S380 phosphorylation and inhibits PI3K/Akt signaling pathway. In the context of IL-7 stimulation, CIGB-300 blocks janus kinase/signal transducer and activator of transcription (JAK/STAT) signaling pathway in T-ALL cells. Altogether, our results strengthen the case for anti-CK2 therapeutic intervention in T-ALL, demonstrating that CIGB-300 (given its ability to circumvent the effects of pro-leukemic microenvironmental cues) may be a valid tool for clinical intervention in this aggressive malignancy.

## 1. Introduction

Acute lymphoblastic leukemia (ALL) is a major childhood cancer and an aggressive hematological malignancy. Patients with ALL of T-cell origin (T-ALL) are considered high risk and, as such, are treated with intensive chemotherapeutic regimens. This has led to very high treatment efficacy, with event-free survival rates at 5 years reaching 90%. However, this success is not without caveats: short- and long-term toxicities are considerable, and relapsed patients have an extremely poor prognosis. Moreover, in adults, therapeutic success is considerably less impressive: only 30–40% of the cases survive long-term. This stresses the need for new therapeutic drugs. Such compounds should selectively target specific genetic lesions and/or pathways connected to T-ALL cell survival and proliferation while sparing normal cells. Major signaling pathways contributing to the expansion and maintenance of T-ALL cells include phosphatidylinositol 3-kinase (PI3K)/Akt, mitogen-activated protein kinase (MAPK), and janus kinase/signal transducer and activator of transcription (JAK/STAT) [1]. Particularly, the PI3K/Akt pathway has been found consistently hyperactivated in roughly 88% of T-ALL clinical samples [2] due to different mechanisms [3,4,5]. Accordingly, an increasing number of targeting agents against components of PI3K/Akt signaling pathway is being investigated in Phase I/II clinical trials for T-ALL treatment [6].

One of the emerging players in T-ALL biology is CK2 (also known as casein kinase 2), a constitutively active serine/threonine protein kinase whose activity accounts for nearly 20% of the human phosphoproteome and is implicated in a myriad of cellular processes [7,8]. The role of CK2 in the maintenance of malignant phenotypes has been extensively studied in solid tumors (reviewed in [9]), and in hematological malignancies (reviewed in [10] and [11]), including in ALL [2,12,13,14,15,16,17,18]. The role of CK2 in T-ALL in particular, with evidence that CK2 accelerates T-cell leukemogenesis in mice, has been recognized for years [19]. CK2 is overexpressed in primary T-ALL cells [2,15], and CK2-mediated phosphorylation of phosphatase and tensin homologue (PTEN) at S380 induces the stabilization of the phosphatase in a non-functional state [20], leading to aberrant PI3K/Akt signaling activation and consequently increased leukemia cell survival and proliferation [2]. This effect may be reinforced by the fact that CK2 phosphorylates Akt at S129, contributing to its full activation [21]. In addition to the impact of CK2 on PTEN and PI3K/Akt-mediated signaling, CK2 can promote B- and T-ALL also via regulation of Ikaros [22]. Moreover, there is evidence for a crosstalk between CK2 and JAK/STAT signaling pathway that can contribute to leukemia cell survival and proliferation [23,24,25], including in response to critical microenvironmental stimuli, such as interleukin-7 (IL-7) [25,26]. 

These findings fostered the exploration of CK2 inhibitors in T-ALL [27], leading to the clear demonstration that pharmacological abrogation of CK2 activity, alone [2,27] or in combination with other targeted therapies [15,18,25,28,29], leads to leukemia cell death in vitro and delays tumor progression in vivo [27]. The therapeutic potential of CK2 inhibitors for T-ALL treatment is illustrated by these studies. However, only CX-4945 (also known as Silmitarsetib [30,31]), and the cyclic peptide CIGB-300 [32] have been tested in cancer clinical trials. CIGB-300 is a peptide chimera containing a cell-penetrating moiety and designed to impair CK2-mediated phosphorylation by binding to the substrate conserved phosphoacceptor sites [32]. This peptide exhibited pro-apoptotic and antitumor effects in different pre-clinical cancer models [33,34,35] and has been tested in Phase I/II clinical trials for the treatment of cervix and lung cancers [36,37,38]. Whereas CX-4945/silmitarsetib has been evaluated in a diversity of T-ALL studies [15,25,27,28], the anti-tumoral effects and mechanisms of action of CIGB-300 in this malignancy remain unaddressed.

Here, we evaluated the impact of CIGB-300 on T-ALL cell viability and proliferation. Taking into consideration the role of IL-7 in promoting T-ALL cell survival and proliferation in vitro [39,40,41,42,43] and T-ALL expansion in vivo [44,45], we also determined the ability of CIGB-300 to target leukemia cells in the presence of IL-7. Furthermore, since other extrinsic signals can contribute to T-ALL cell survival, we evaluated whether murine OP9-delta-like 1 (DL1) stromal cells counteracted the cytotoxic effect of CIGB-300 in co-culture experiments. We demonstrate that CIGB-300 reduces the viability and proliferation of T-ALL cells even in the context of IL-7- or stromal-mediated supportive signals. While CIGB-300 binds to and impairs the phosphorylation of nucleophosmin 1 (B23/NPM1), previously described as the molecular target of CIGB-300 in solid tumor cells [46], the anti-leukemic effects of CIGB-300 in malignant T cells appear to be explained by direct CK2 enzyme inhibition.

## 2. Results

### 2.1. CIGB-300 Negatively Impacts Viability and Proliferation of T-ALL Cells

**Figure 1:**
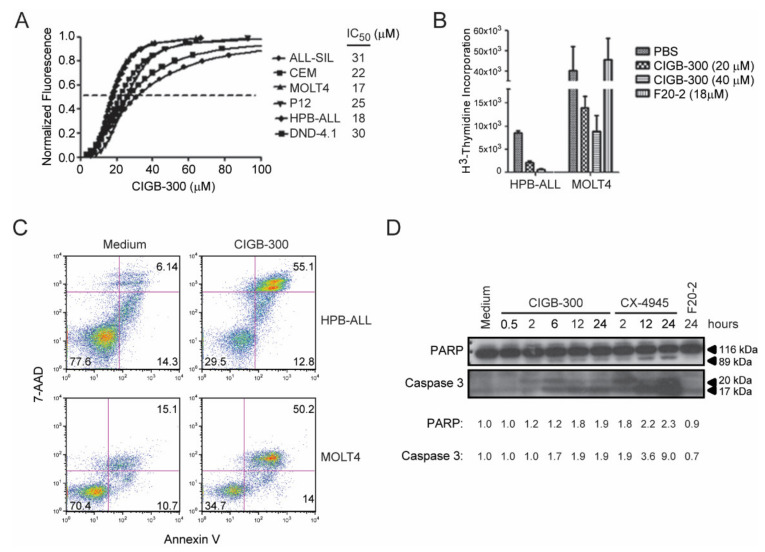
CIGB-300 negatively impacts the viability and proliferation of T-ALL cells. The indicated T-ALL cell lines were cultured for 72 h with increasing concentrations of CIGB-300. (**A**) Viability/proliferation was evaluated by Alamar Blue. (**B**) Proliferation was measured by ^3^H-thymidine incorporation. (**C**) Viability of HPB-ALL and MOLT4 cells was evaluated by Annexin V-APC conjugate/ 7-aminoactinomycin D (7-AAD) staining after 16 h in the presence of CIGB-300 at 18 µM. (**D**) Detection of poly (ADP-ribose) polymerase (PARP) and caspase-3 cleavage was determined in HPB-ALL cells by western blot after treatment with CIGB-300 (18 µM), CX-4945 (12 µM) or control peptide F20-2 (18 µM) for the indicated time intervals. Relative densitometry analysis values of cleaved PARP and cleaved caspase 3 (17 kDa) bands, normalized to medium, are indicated. Results from (**A**,**B**) represent mean ± SD of 3 replicates, while those from (**C**,**D**) are representative of 2 independent experiments (2 replicates each).

To evaluate the effect of CIGB-300 on the viability of T-ALL cells we selected six well-established cell lines representing different oncogenic subgroups and stages of maturation (Table 1). CIGB-300 had a dose-dependent inhibitory effect on the viability and/or proliferation of all the tested cell lines, with half-inhibitory concentrations (IC_50_) ranging from 17 to 31 µM (mean IC_50_ = 23.8 µM) as measured using Alamar Blue (Figure 1A). The impact of CIGB-300 on the proliferation of HPB-ALL and MOLT4 cells was discriminated by the evaluation of ^3^H-thymidine incorporation. CIGB-300 inhibited proliferation of both cell lines in a dose-dependent manner (Figure 1B), whereas a control peptide (F20-2), containing only the cell-penetrating moiety of CIGB-300 [32], did not have any significant impact (Figure 1B). Furthermore, CIGB-300 promoted T-ALL cell apoptosis as measured by Annexin V-APC conjugate and 7-aminoactinomycin D (7-AAD) staining (Figure 1C) and caspase 3 and poly (ADP-ribose) polymerase (PARP) cleavage (Figure 1D). As expected, the negative control peptide F20-2 did not upregulate caspase 3 or PARP cleavage, and the CK2 small molecule inhibitor CX-4945 induced a strong cleavage (Figure 1D). 

### 2.2. CIGB-300 Impairs CK2-Mediated Phosphorylation of PTEN, Akt and B23/NPM1

**Figure 2:**
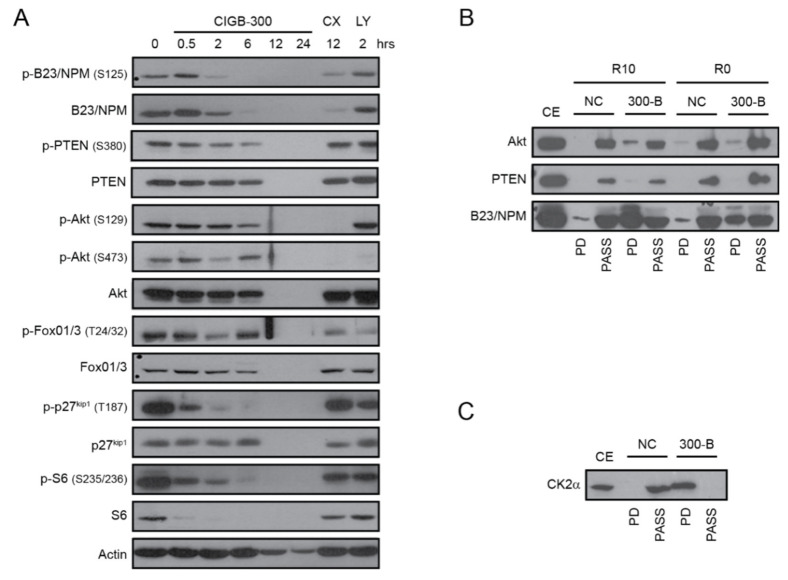
Effect of CIGB-300 on CK2-mediated phosphorylation of Akt, PTEN, B23/NPM1 and their potential interactions in HPB-ALL cells. (**A**) Phosphorylated and total protein levels of CK2 substrates Akt, PTEN, and B23/NPM1, and PI3K/Akt pathway members, in HPB-ALL cells incubated with either 18 µM of CIGB-300 or the inhibitors CX-4945 (CX, 12 µM) and LY294002 (LY, 10 µM) for the indicated time. Actin was used as loading control. (**B**) Immunoblots from pull-down fractions using CIGB-300 conjugated to biotin as bait to capture interacting proteins. HPB-ALL cells were incubated for 1 h with biotin-tagged peptide (50 µM), subsequently lysed and submitted to SDS-PAGE and antibody detection as indicated. The experiments were performed in RPMI with or without 10% FBS (R10 or R0, respectively), as indicated. (**C**) Pull-down performed with cellular lysates of HPB-ALL cells as indicated above. CE, Cellular Extract. PD, Pull-Down fractions. PASS, flow-through fractions. NC, negative control (cellular lysate from HPB-ALL cells incubated with vehicle).

The effect of CIGB-300 on the phosphorylation of two CK2 direct substrates involved in T-ALL biology (PTEN and Akt), as well as downstream signaling players forkhead box O1/3 (FoxO1/3), cyclin-dependent kinase inhibitor 1B (CDKN1B/p27^kip1^) and ribosomal protein S6 (S6), was evaluated in HPB-ALL cells. In addition, we analyzed the levels of B23/NPM1, a known molecular target of CIGB-300 in solid tumor cells [46]. As we previously reported, CIGB-300 blocked the phosphorylation of S125 residue on B23/NPM1 after 2 h of incubation (Figure 2A). CIGB-300 also impaired the phosphorylation of CK2-phosphoaceptor residues on PTEN (S380) and Akt (S129) after 6 h (Figure 2A), similar to our previous findings on chronic lymphocytic leukemia (CLL) cells [47]. Accordingly, phosphorylation of PI3K/Akt downstream targets FoxO1/3 (T24/32), p27^kip1^ (T187), and S6 (S235/236) were also inhibited by CIGB-300 (Figure 2A). As expected, CX-4945 inhibited Akt phosphorylation at both S129 and S473, whereas the PI3K inhibitor LY294002 did not affect phosphorylation at S129 (directly mediated by CK2) but abrogated S473 phosphorylation, which depends on the balance between PI3K and PTEN activities (Figure 2A).

To determine whether CIGB-300 directly interacts with PTEN, Akt or B23/NPM1, we next performed pull-down experiments in HPB-ALL cells. In both experimental conditions (medium with serum, R10, and without serum, R0) we detected a clear interaction between CIGB-300 and B23/NPM1, a weak interaction with Akt, and no interaction with PTEN (Figure 2B). The lack of interaction between CIGB-300 and PTEN, as well as the mild interaction with Akt, was intriguing, given the clearly inhibitory effects of CIGB-300 on PTEN S380 phosphorylation (which is known to be directly mediated by CK2) and on phosphorylation of Akt and its downstream targets (Figure 2A). Therefore, we hypothesized that CIGB-300 may directly bind to CK2 and thereby inhibit CK2 activity (towards at least the substrates we analyzed). Our pull-down experiments confirmed that CIGB-300 can bind to CK2α catalytic subunit (Figure 2C).

### 2.3. B23/NPM1 Is not a Major Target for CIGB-300 in T-ALL Cells

**Figure 3:**
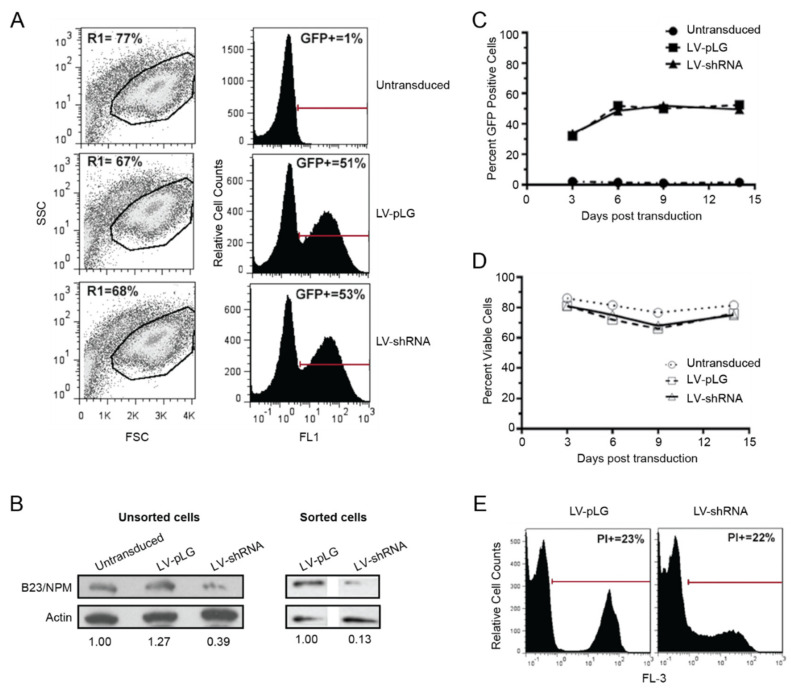
Silencing of *NPM1* does not mimic the effects of CIGB-300 on HPB-ALL cells. HPB-ALL cells were transduced with mock vector or shRNA against *NPM1* and analyzed by flow cytometry at indicated time intervals. (**A**) Percentage of transduced cells on live-cell populations, as identified by forward scatter (FSC) × side scatter (SSC) discrimination (R1 gate in dot plots on the left), was determined by analysis of GFP expression at day 9 post-infection (histograms on the right). Percentage of GFP-positive cells was calculated using untransduced cells as a negative control. (**B**) Immunoblot analysis of transduced cells showing B23/NPM1 protein knock down in total unsorted population (~60% decrease) and sorted GFP-positive cells (~90% decrease). Actin was used as a loading control. Relative densitometry analysis values of B23/NPM bands normalized to actin and then to either untransduced (unsorted cells) or LV-pLG (sorted cells) lanes are indicated. (**C**,**D**) Analysis of (**C**) GFP expression within the live cell population and (**D**) viability of HPB-ALL cells at the indicated time points after transduction. (**E**) Cytotoxic effect of CIGB-300 (18 µM) on LV-pLG or LV-shRNA *NPM1* transduced HPB-ALL cells as assessed by propidium iodide (PI) staining and flow cytometry analysis.

Given the importance of B23/NPM1 inhibition for the effect of CIGB-300 in solid tumors, we next investigated whether a similar mechanism could account for CIGB-300 anti-leukemia effects by silencing *NPM1* in HPB-ALL cells. The cells were incubated with lentiviral particles (LV) expressing an shRNA against the 3’-UTR of *NPM1* and the infected, green fluorescent protein (GFP)-positive, population was examined by flow cytometry for at least two weeks (Figure 3). HPB-ALL cells transduced with either empty vector (pLG) or *NPM1* shRNA showed no difference in viability (Figure 3A), despite a knock down of B23/NPM1 protein levels of at least 60% (Figure 3B). After 9 days of LV infection, roughly 50% of transduced HPB-ALL cells were GFP-positive, irrespective of the condition (Figure 3A,C), suggesting that decreased B23/NPM1 expression did not negatively impact T-ALL cell fitness. Indeed, HPB-ALL cells were followed for two weeks after transduction with empty vector or *NPM1* shRNA and consistently presented similar levels of GFP expression (Figure 3C) and viability (Figure 3D) in both conditions. Finally, the effect of CIGB-300 on the viability of HPB-ALL cells was not affected by *NPM1* silencing (Figure 3E). Overall, these results indicate that, despite the binding between the two occurring in T-ALL cells, B23/NPM1 inhibition does not appear to have a critical role in the anti-leukemia effects of CIGB-300.

### 2.4. The Effects of CIGB-300 on T-ALL Cells Are not Reversed by IL-7 Stimulation or Stromal Support

**Figure 4:**
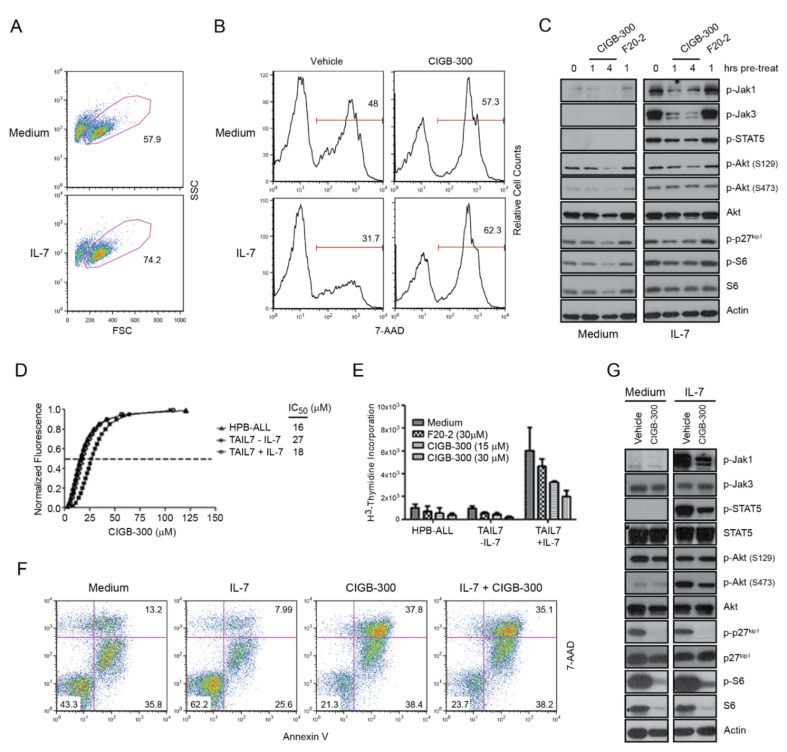
CIGB-300 decreases the viability and proliferation of T-ALL cells irrespectively of IL-7-stimulation. (**A**) Evaluation of HPB-ALL cell viability by FSC × SSC discrimination and flow cytometry analysis after stimulation with IL-7 (50 ng/mL) for 48 h. (**B**) Evaluation of HPB-ALL cell viability by 7-AAD staining and flow cytometry analysis, after 48 h of incubation with 18 µM of CIGB-300 in the presence or absence of IL-7 (50 ng/mL). (**C**) Immunoblot analysis of PI3K/Akt and JAK/STAT signaling pathway activation. HPB-ALL cells cultured for 24 h in low serum (R1) were pre-incubated for 15 min with the CIGB-300 or F20-2 (18 µM) and then incubated with IL-7 (50 ng/mL) or medium for 45 min or 3h 45min (for a total of 1 or 4 h). Control cells were (pre)incubated with vehicle alone for a total of 1h. (**D**,**E**) TAIL7 cells were incubated with different concentrations of CIGB-300 with or without IL-7 (20 ng/mL) and cell viability and proliferation were measured by Alamar Blue (**D**) and ^3^H-thymidine incorporation (**E**) at 48 h. (**F**) Annexin V-APC/7-AAD dot plots of HPB-ALL cells treated as above and incubated with CIGB-300 at 18 µM for 16 h. (**G**) Effect of CIGB-300 on the phosphorylation of indicated proteins in TAIL7 cells stimulated or not with IL-7 (20 ng/mL). The cells were pre-incubated during 15 min with CIGB-300 (18 µM) or vehicle in medium (R1), and then IL-7 or medium were added to complete 1 h of incubation.

We next evaluated whether the effects of CIGB-300 on viability and proliferation of T-ALL cells could be counteracted by IL-7-mediated signals, which are known to prevent apoptosis and promote T-ALL growth in vitro and in vivo [26]. As expected [48], addition of IL-7 to the culture medium increased the survival of HPB-ALL cells (Figure 4A,B). However, the pro-survival effect of IL-7 was completely blocked by CIGB-300 (Figure 4B). At the molecular level, CIGB-300 downregulated both basal and IL-7-mediated Akt, p27^kip1^, and S6 phosphorylation in HPB-ALL cells (Figure 4C). Likewise, IL-7-mediated activation of JAK/STAT pathway, measured by JAK1, JAK3, and STAT5 phosphorylation, was blocked by pre-treatment with CIGB-300 (Figure 4C).

To corroborate these findings, we next evaluated the impact of CIGB-300 on the T-ALL primary leukemia-like IL-7-dependent cell line TAIL7. Alamar Blue, ^3^H-thymidine and Annexin V/7-AAD staining assays demonstrated that CIGB-300 reduced the viability and proliferation of TAIL7 cells irrespectively of IL-7 stimulation (Figure 4D–F). Similar to the results in HPB-ALL cells, CIGB-300 abrogated JAK1/STAT5 signaling upon IL-7 stimulation, and downregulated the phosphorylation of Akt, p27^kip1^, and S6 in TAIL7 cells (Figure 4G).

**Figure 5:**
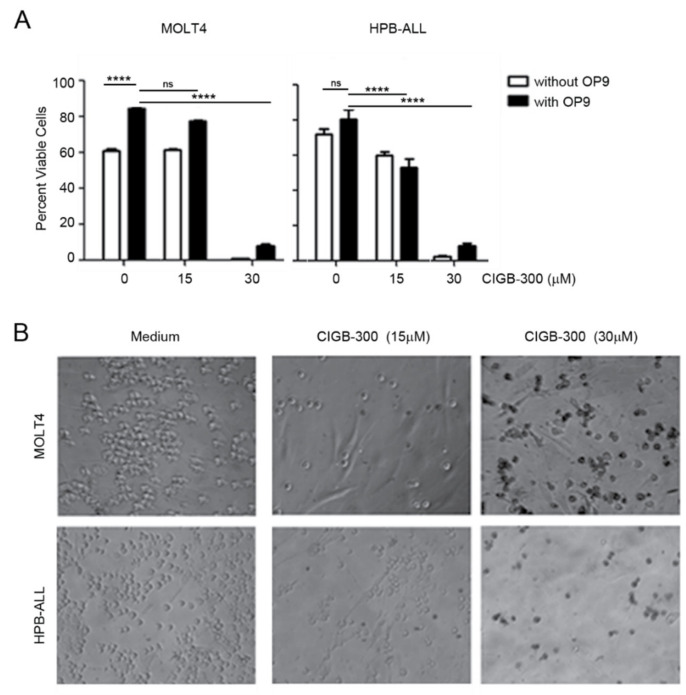
CIGB-300 decreases the viability of T-ALL cells co-cultured with OP9-hDL1 stromal cells. MOLT4 and HPB-ALL cells were cultured alone or with OP9-hDL1 cells for 48 h with or without the indicated concentrations of CIGB-300. Cell viability was determined by (**A**) Annexin V-APC/7-AAD staining and flow cytometry, or (**B**) Trypan blue staining and light microscopy. Results shown in (**A**) are mean ± SD of triplicates. At least two independent experiments were performed on each case. ns, not significant; **** *p*-value < 0.0001.

Because IL-7 is not the only external cue that may counteract the impact of CIGB-300, we next evaluated whether stromal support inhibited CIGB-300 anti-T-ALL effects. We co-cultured MOLT4 and HPB-ALL cells with murine OP9 cells expressing human Notch ligand Delta-like 1 (OP9-hDL1) and assessed the pro-apoptotic effects of CIGB-300. Annexin V/7-AAD staining showed that a potent dose of CIGB-300 (IC_85_) abrogated leukemia cell viability irrespectively of the survival signals provided by stromal cells (Figure 5A). Although we still observed some mild OP9-DL1 supportive effects at this peptide dose, they were not sufficient to reverse CIGB-300 cytotoxic effect (Figure 5A). Moreover, bright field microscopy analysis of Trypan Blue staining of MOLT4 and HPB-ALL co-cultured with OP9-hDL1 cells and treated with CIGB-300 for 48 h showed a dose-dependent cytotoxic effect of the peptide specifically on leukemia cells but not on the stromal monolayer (Figure 5B).

### 2.5. Primary T-ALL Patient Cells Are Sensitive to CIGB-300 even in the Presence of IL-7

**Figure 6:**
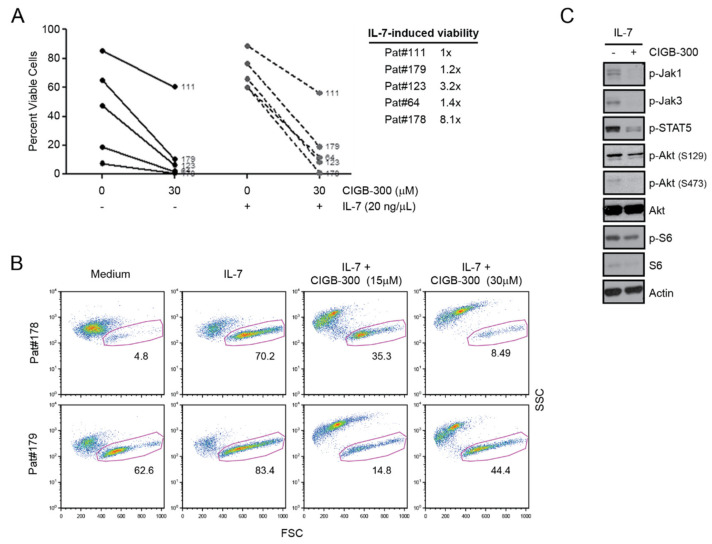
Primary T-ALL patient cells are sensitive to CIGB-300 even in the presence of IL-7. (**A**,**B**) T-ALL cells isolated from peripheral blood or bone marrow of pediatric patients at diagnosis were incubated with or without IL-7 and the indicated concentrations of CIGB-300 for 48 h and subsequently analyzed by flow cytometry to determine cell viability. (**A**) Percentage of viable cells for each patient sample in the indicated conditions. Impact of IL-7 stimulation on primary T-ALL cell viability is shown as fold change compared to unstimulated cells. (**B**) FSC × SSC dot plots of two representative primary T-ALL samples. Percentage of viable cells is indicated. (**C**) Immunoblots with indicated antibodies using lysates from primary T-ALL cells from Patient #179. Cells were pre-incubated for 15 min with or without CIGB-300 (30 µM) and then IL-7 (20 ng/mL) was added for 45 min.

To further explore the therapeutic potential of CIGB-300 we next cultured primary T-ALL cells collected from pediatric patients at diagnosis (Table 1) for 48 h with or with IL-7 in the presence or absence of CIGB-300. The peptide decreased the viability of all tested primary samples in medium without IL-7 (Figure 6A). Addition of IL-7 provided a viability push in 4 of the 5 tested T-ALL samples (Figure 6A). However, this effect was reversed by CIGB-300 (Figure 6A), suggesting that CIGB-300 should have an anti-T-ALL effect even in IL-7-rich in vivo niches. The cytotoxic effect of CIGB-300 in the presence of IL-7 correlated with the ability of CIGB-300 to block IL-7-mediated JAK/STAT pathway activation, as measured by JAK1, JAK3 and STAT5 phosphorylation, and to downregulate IL-7-mediated PI3K/Akt pathway activation, as measured by Akt and S6 phosphorylation (Figure 6C).

## 3. Discussion

We have previously shown that CK2 inactivates PTEN and thereby leads to PI3K/Akt signaling pathway hyperactivation and consequent T-ALL cell survival in vitro [2] and in vivo [27]. We have also demonstrated that similar mechanisms of CK2-mediated PTEN inactivation exist in B-ALL and CLL and are of relevance for leukemia cell maintenance [12,47,49]. Moreover, CK2 is relevant in biology, and resistance to therapy, of acute myeloid leukemia (AML) and chronic myeloid leukemia (CML) [11,50,51,52,53]. Thus, there is a clear rationale to explore the use of CK2 inhibitors in the clinical setting in different leukemias, including T-ALL. However, to our knowledge, only two molecules targeting CK2-mediated signaling have reached the clinical stage, namely CX-4945/Silmitasertib and CIGB-300 [31,36].

Contrary to conventional ATP-competitive inhibitors that target the catalytic subunits of CK2, CIGB-300 impairs CK2-mediated phosphorylation by direct binding to a conserved phosphoacceptor domain [32]. Previous preclinical and clinical studies have demonstrated the antineoplastic potential of CIGB-300 in solid tumors [33,34,35,36,37,38]. However, the exploration of this anti-CK2 peptide in hematological cancers is still limited [47]. Here, we pre-clinically evaluated the impact of the clinical-grade peptide-based CK2 inhibitor CIGB-300 on T-ALL cell lines and primary cells belonging to different oncogenic subgroups and blocked at distinct stages of maturation. Our studies demonstrated that CIGB-300 had a broad, dose-depended negative impact on the viability and proliferation of T-ALL cells.

To identify potential CK2 substrates that can be targeted by CIGB-300 in T-ALL cells, we conducted signaling experiments with antibodies directed against CK2 phosphoacceptor sites in Akt (S129) and PTEN (S380), as well as against B23/NPM1, previously recognized as a major target for CIGB-300 in solid tumors [46]. Our results showed that CIGB-300 impairs the phosphorylation of Akt S129 and PTEN S380 after short term incubation. Accordingly, the phosphorylation levels of downstream PI3K/Akt pathway members FoxO1/3, p27^kip1^, and S6 also decreased, temporally preceding the cleavage of the apoptotic marker caspase-3. CIGB-300 also reduced the phosphorylation and total protein levels of B23/NPM1. 

Subsequent pull-down experiments indicated that among the CK2 substrates evaluated, only B23/NPM1 significantly interacts with CIGB-300 in HPB-ALL cells. However, silencing of *NPM1* produced no significant effect on viability or proliferation of T-ALL cells, and did not potentiate the cytotoxic effect of CIGB-300. Altogether, these results indicated that B23/NPM1 is not a major functional target of CIGB-300 in T-ALL cells.

Although CIGB-300 did not directly interact with PTEN and only mildly with Akt, it clearly impaired their phosphorylation in HPB-ALL cells. We clarified these puzzling findings by revealing that CIGB-300 can directly interact with the CK2α catalytic subunit of the enzyme and inhibit its activity in T-ALL cells, as demonstrated by the inhibition of Akt S129 phosphorylation, a surrogate marker for CK2 enzymatic activity in cells [21]. These observations explain the inhibition of Akt S473 and PTEN S380 phosphorylation (and consequent inhibition of some of the downstream targets FoxO1/3, S6, and p27^kip1^) without the requirement for direct interaction between CIGB-300 and the two CK2 substrates. In other words, besides its known binding ability to CK2 phosphoacceptor sites, CIGB-300 can also directly bind to CK2 and thereby prevent its activity towards (all or some of) its substrates. Of note, our recent findings using cell-free CK2 kinase assays and lung cancer cells also support a direct impairment of CK2 activity by CIGB-300 [54].

IL-7, produced in the bone marrow and thymus, is a major microenvironmental signal promoting T-ALL cell expansion [26,39,42,43,44,48,55,56,57]. We have previously found that CK2 activity is mandatory for optimal IL-7/IL-7R-mediated signaling. Using CX-4945, we demonstrated that CK2 pharmacological inhibition impaired JAK/STAT5 and PI3K/Akt signaling pathway activation triggered by IL-7 or by *IL7R* mutational activation [58] and consequently impaired the pro-leukemia effects of IL-7 [25]. We now showed that CIGB-300 inhibits not only basal but also IL-7-mediated signaling and survival/proliferation in the primary-like IL-7-dependent TAIL7 cell line [41] and, most importantly, in primary patient samples. This is reassuring from a clinical perspective, since there is evidence that IL-7 promotes T-ALL cell expansion in patients [44] and is known to confer resistance to other treatments, such as glucocorticoids [59,60]. 

CK2 can regulate components of the JAK/STAT signaling complex, and JAKs are members of the CK2 interactome [23,24]. Similar to CX-4945 [25], CIGB-300 impaired JAK/STAT and PI3K/Akt activation triggered by IL-7. The rapid and strong inhibition of phosphorylation of JAK1 and JAK3, as well as of STAT5, might be explained by the trans-activating regulatory protein (TAT)-mediated internalization mechanism [61] used by CIGB-300. CK2 has been involved in the process of receptor internalization through the formation of clathrin-coated pits [62], whereas IL-7 promotes IL-7R internalization by the same endocytic pathway, which is required for optimal IL-7-mediated signaling [63]. It is thus possible that CIGB-300, CK2, and the IL-7/IL-7R receptor complex could “prematurely meet” at the cell surface during the first steps of peptide internalization, thus accounting for the observed downstream inhibition of JAK/STAT and PI3K/Akt signaling pathways, once T-ALL cells are in the presence of IL-7. The fact that CK2 is able to physically interact with IL-7R [25] is in line with this possibility, which warrants further investigation.

Other environmental cues produced by stromal cells in the leukemic niche also partake in the onset and maintenance of T-ALL [3,64]. The OP9-DL1 co-culture system has emerged as a valuable in vitro model for T-cell development but also to study the relevance of extracellular signals for leukemia cell survival [65,66]. In addition to Notch signaling, these murine stromal cells produce a cocktail of cytokines that promotes cell survival, proliferation, and differentiation of T-cells, hence mimicking the in vivo microenvironment [67]. Our demonstration that CIGB-300 reduces the viability of T-ALL cells in co-culture with OP9-DL1 cells reinforces the potential of using CIGB-300 for therapeutic purposes in the clinical setting. 

## 4. Materials and Methods 

### 4.1. Cell Lines and Patient Samples

Human T-ALL cell lines HPB-ALL, MOLT4, DND-4.1, ALL-SIL, CEM, and P12 were cultured in RPMI 1640 medium (Invitrogen, Carlsbad, CA, USA) supplemented with 10% (vol/vol) heat-inactivated fetal bovine serum (FBS, Invitrogen, Carlsbad, CA, USA), 10 mM penicillin-streptomycin solution, and 2mM L-glutamine (hereafter referred to as R10 medium). Primary-like IL-7-dependent cell line TAIL7 was maintained on IL-7 continuous culture and routinely submitted to viability and proliferation assays to verify IL-7 responsiveness and dependency [41]. Primary T-ALL cells were collected at the Pediatrics Service of Instituto Português de Oncologia de Lisboa Francisco Gentil, Lisbon, Portugal, from the peripheral blood and/or bone marrow of pediatric patients with high leukemia involvement (85–100%). Informed consent was obtained for all sample collections and the study conducted after institutional review board approval by Gabinete de Investigação Clínica, in accordance with the Declaration of Helsinki. Samples were enriched by density centrifugation over Ficoll-Paque (GE Healthcare, Chicago, IL, USA) and subsequently processed as described [42]. All cell cultures were maintained at 37 °C in 5% CO_2_.

### 4.2. Alamar Blue Assay

Cell viability/proliferation was determined using Alamar Blue assay (Life Technologies, Carlsbad, CA, USA). Briefly, T-ALL cells were seeded in flat-bottom 96-wells plates as 10^6^ cells/mL in R10 and a curve of serial dilutions (1:2) of CIGB-300 (100–3.12 µM) were added. After 72 h, Alamar Blue was added in an amount equal to 10% of the culture volume. Cells were further incubated at 37 ℃, 5% CO_2_ for 2–4 h. Fluorescence was measured at wavelengths of 530 nm excitation and 580 nm emission in an Infinite M200 plate reader (Tecan, Durham, NC, USA). The IC_50_ was estimated from the fitted dose-response curves using the software Calcusyn (Biooft, Cambridge, UK) [68].

### 4.3. Assessment of Cell Viability

Viability of T-ALL cells was determined by flow cytometry analysis in a FACSCalibur instrument (Becton Dickinson, Franklin Lakes, NJ, USA), after staining with Annexin V-APC (eBioscience, San Diego, CA, USA) and 7-AAD.

### 4.4. ^3^H-Thymidine Incorporation Proliferation Assay

After T-ALL cells were exposed to selected concentrations of CIGB-300 or CX-4945 compound, the DNA synthesis was assessed using a ^3^H-thymidine-based protocol. ^3^H-thymidine (1 Ci/well [0.037MBq/well], PerkinElmer, Waltham, Massachusetts, USA) was added for 16 h prior to cell harvest and measured by using a liquid scintillation counter. Proliferation index was calculated as (mean cpm for each experimental condition)/(mean cpm for medium alone).

### 4.5. Signaling Experiments

Exponentially growing HPB-ALL cells were washed and seeded in 6-well cell culture plates as 2 × 10^6^ cells/mL using serum-free RPMI (hereafter referred to as R0). Then, selected concentrations of CIGB-300, CX-4945, or F20-2 negative control peptide were added in dose-response experiments for 2 h or incubated during 0.5, 2, 6, 12, and 24 h in kinetic experiments. In some experiments the selective PI3K inhibitor LY294002 (10 µM; Calbiochem, San Diego, CA, USA) was used. Signaling experiments on TAIL7 cells were conducted after starving the cells during 24 h in RPMI with 1% FBS (Invitrogen, Carlsbad, CA, USA). Subsequently, cells were washed, resuspended as 2 × 10^6^ cells/mL in RPMI with 5% FBS, and incubated with CIGB-300 (18 µM) or CX-4945 (12 µM) for 1 h. In selected experiments, 15 min after addition of the drugs, 20 ng/mL of recombinant IL-7 (Peprotech, Rocky Hill, NJ, USA) were added and incubated for 45 min. Finally, signaling experiments on IL-7-responsive HPB-ALL cells were conducted on RPMI with 1% FBS (R1) after 24 h starvation by adding recombinant IL-7 up to a final concentration of 50 ng/mL.

### 4.6. Western Blot Analysis

Cell lysates from signaling experiments were prepared as described [41], and equal amounts of protein (50 µg/sample) were resolved by 12% sodium dodecyl sulfate-polyacrylamide gel electrophoresis (SDS-PAGE), transferred onto nitrocellulose membranes, and immunoblotted with the following antibodies: p-Akt (T308), Akt, p-PTEN (S380), PTEN, p-FoxO1/3 (T24/32), FoxO1/3, p-S6 (S235/236), S6, p-JAK1 (Y1022/1023), p-JAK3 (Y980/981) (Cell Signaling Technology, Danvers, MA, USA); p-Akt (S473), STAT5a, Caspase 3, Actin (Santa Cruz Biotechnology, Dallas, TX, USA); p-B23/NPM1 (S125), B23/NPM1 (Abcam, Cambridge, UK); p-Akt (S129) (ABGent, San Diego, CA, USA); p-P27^kip^ (T187) (Zymed, San Francisco, CA, USA); P27^kip1^ (BD Pharmingen); p-STAT5a/b (Y694/699) (Upstate Biotechnology, Lake Placid, NY, USA); PARP (Novus Biologicals, Littleton, CO, USA) and CK2A1/A2 (BD Transduction Laboratories, San Diego, CA, USA). After immunoblotting with antibodies, detection was performed by incubation with horseradish peroxidase-conjugated anti–mouse, anti–rabbit (Promega, Madison, WI, USA), or anti–goat (Santa Cruz Biotechnology) immunoglobulin (IgG; 1:5000 dilution), and developed by chemo luminescence (Amersham Pharmacia Biotech, Little Chalfont, UK). Densitometry analysis was performed using ImageJ software and integrated density values of each band were used to calculate values relative to the loading control in each lane/condition. Where indicated relative values were then normalized to the respective control conditions, or, in the case of pull-down experiments (see Section 4.7 Pull-Down), to whole-cell extracts. Stripping and reprobing of the immunoblots were done as described [69]. Detailed information about western blot can be found in Figures S1–S7.

### 4.7. Pull-Down

Pull-down experiments were performed essentially as described earlier [46]. Briefly, 20 × 10^6^ cells were seeded in 10 mL of R0 or R10 and incubated for 1 h with 50 µM of CIGB-300 conjugated to biotin. Afterward, the cells were lysed in hypotonic PBS solution (0.1×) containing 1 mmol/L of DTT (Sigma, St. Louis, MO, USA) and complete protease inhibitor (Roche, Basel, Switzerland) by eight freeze-thaw (liquid nitrogen/37 °C) cycles. Then, the cellular lysate was cleared by centrifugation and 200 μg of total protein were added to 30 μL of pre-equilibrated streptavidin-sepharose matrix (Amersham Pharmacia Biotech, Little Chalfont, UK). After 1 h of incubation at 4 °C, CIGB-300´s-interacting proteins were eluted, resolved in an SDS-PAGE gel, and transferred onto nitrocellulose membranes to perform western blot experiments. For CK2 pull-down, CIGB-300 conjugated to biotin was incubated for 1 h at 4 °C with 200 µg of total protein lysates and then treated as above.

### 4.8. Lentiviral Infection

The transcriptional unit for *NPM1* knockdown was amplified from a pSilencer1.0–U6 siRNA Expression Vector (Ambion, Austin, TX, USAUSA) containing a shRNA sequence against the 3′UTR region of *NPM1* isoform 1 (TRCN0000062268, *NPM1* MISSION shRNA, Sigma). The PCR product was then digested and inserted into XbaI restriction site of the lentiviral transfer plasmid pLGW [70]. This plasmid includes a GFP reporter gene under the SV40 promoter, and HIV-based third-generation lentiviral vector elements to produce infective particles once co-transfected with packaging plasmids pLP1, pLP2 and pLP/VSVG in HEK293T cells (ViraPower Lentiviral Packaging Mix, Thermo Fisher Scientific, Waltham, MA, USA). Virus production and subsequent titration of viral particles were performed according to previously described protocols [71]. HPB-ALL cells were infected by spinoculation at a 10x multiplicity of infection (MOI10) [72]. Briefly, the cells were seeded as 0.5 × 10^6^ cells/mL, in preconditioned medium, with polybrene (8 µg/mL) plus the viral supernatant, and then centrifuged at 800× *g* for 30 min at 32 °C. On the next day, fresh complete medium was added and the cells allowed to recover for an additional 48 h. HPB-ALL cells were evaluated by flow cytometry analysis for confirmation of GFP expression as a measure of transduction efficiency (Cyflow, Partec, Germany).

### 4.9. Co-Culture Experiments

OP9-hDL1, a bone-marrow-derived murine stromal cell line that ectopically expresses the Notch ligand Delta-like 1 (Dl1) [73] was cultured in Dulbecco’s Modified Eagle’s Medium (DMEM) medium supplemented with 10% FBS for several days until a healthy cell monolayer was evident. Then, 2.5 × 10^4^ stromal cells were seeded onto 48-well culture plates (5 × 10^4^ cells/mL) and incubated for 24 h to allow the cells to attach. Wells were then washed twice with pre-warmed PBS and 10^6^ cells/mL of HPB-ALL or MOLT4 cells were added, with or without CIGB-300 (15 and 30 µM), to each well. After 48 h of incubation, the effect of CIGB-300 on T-ALL cell viability was assessed by Annexin V-APC and 7-AAD staining and flow cytometry analysis and/or by Trypan Blue staining and bright field microscopy evaluation of leukemic cells still attached to OP9-hDL1 cell monolayer.

### 4.10. Statistical Analysis

Differences between groups were calculated one-way ANOVA with Tukey’s multiple comparisons post-test. The analyses were done using GraphPad Prism version 4.00 (GraphPad Software, San Diego, CA, USA) for Windows. Differences were considered significant for a *p*-value < 0.05.

## 5. Conclusions

**Figure 7:**
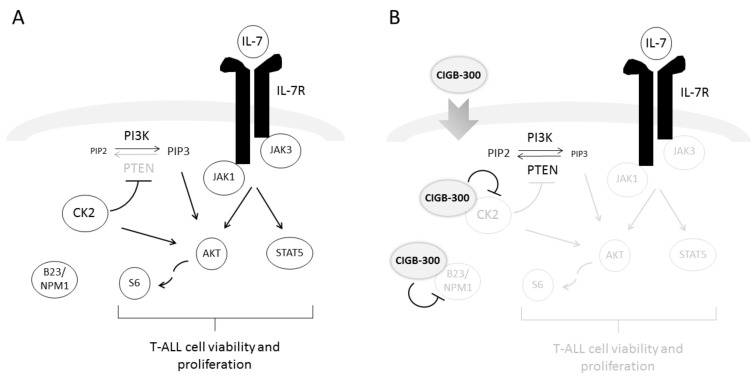
Model of CIGB-300-mediated effects on T-ALL cells. (**A**) T-ALL cell viability and proliferation relies on cell-intrinsic signals and extracellular cues such as IL-7, which activate PI3K/Akt/mTOR and JAK/STAT pathways. CK2 phosphorylates and thereby inactivates PTEN leading to activation of PI3K/Akt/mTOR signaling [2]. This effect is reinforced by CK2-mediated direct phosphorylation of Akt [21]. In addition, CK2 binds to IL-7R (this interaction is not shown in the cartoon), which is essential for IL-7-mediated maximal signaling [25]. (**B**) In the present study, we showed that CIGB-300, a plasma membrane-crossing CK2 peptide antagonist [32], binds to and inhibits B23/NPM1 (which does not appear to be critical for CIGB-300 anti-T-ALL effects) and to CK2. The effects of CIGB-300 in T-ALL cells appear to be mediated mainly by its ability to inhibit CK2 activation, which results in reversion of cell-intrinsic and IL-7-mediated activation of PI3K/Akt/mTOR pathway as well as abrogation of IL-7-mediated JAK/STAT signaling. As a consequence of these effects, T-ALL cells enter apoptosis and no longer proliferate.

In summary, we demonstrate that the clinical-grade cell-penetrating peptide CIGB-300 is able to block proliferation and induce cell death of both cell lines and primary T-ALL patient cells, even in the presence of leukemia-supportive signals such as IL-7 or stromal cell co-culture. We show that CIGB-300 does so by directly binding to and inhibiting CK2 activity, consequently preventing CK2-mediated phosphorylation of PTEN and Akt, and downregulating the activation of pro-survival PI3K/Akt and JAK/STAT signaling pathways (Figure 7). Our studies reveal that the CIGB-300 may be a valid tool for therapeutic intervention in T-ALL.

## Figures and Tables

**Table 1:** Oncogenic subgroup and immunophenotype of Acute lymphoblastic leukemia of T-cell origin (T-ALL) cell lines and primary patient cells collected at diagnosis.

		Subgroup	Immunophenotype
T-ALL cell lines	ALL-SIL	TLX1	CD3− CD4+ CD8+
	CEM	TAL1	CD3− CD4+ CD8−
	MOLT4	TAL1	CD3− CD4+ CD8+
	P12-ICHIKAWA	LMO2	CD3− CD4+ CD8−
	HPB-ALL	TLX3	CD3+ CD4+ CD8+
	DND4.1	TLX3	CD3+ CD4+ CD8−
Primary T-ALL samples	Pat#64	LMO2	CD3− CD4+ CD8+
	Pat#111	LMO2-LYL1	CD3− CD4− CD8−
	Pat#123	ND ^1^	CD3− CD4− CD8−
	Pat#178	TAL1	CD3− CD4+ CD8+
	Pat#179	ND ^1^	CD3− CD4− CD8−

^1^ ND—not determined.

## Supplementary Materials

The following are available online at https://www.mdpi.com/2072-6694/12/6/1377/s1, Figure S1: Non-cropped images of the immunoblots shown in Figure 1D, Figure 2A–C, Figure 3B, Figure 4C,G and Figure 6C; Figure S2: Densitometry analysis values for Figure 2A–C, Figure 4C,G and Figure 6C.
