# Supplementary material for: Clinical-Grade Peptide-Based Inhibition of CK2 Blocks Viability and Proliferation of T-ALL Cells and Counteracts IL-7 Stimulation and Stromal Support
Source: Cancers (Basel). 2020 May 27;12(6):1377. doi: 10.3390/cancers12061377 (PMC7352628; doi:10.3390/cancers12061377)
Supplement: Supplementary file 1 [file cancers-12-01377-s001.pdf]

cancers

MDPI

Supplementary Materials

Clinical-Grade Peptide-Based Inhibition of CK2 Blocks Viability and Proliferation of T-ALL Cells and Counteracts IL-7 Stimulation and Stromal Support

Yasser Perera, Alice Melão, Ailyn de la Caridad Ramón, Dania Vázquez, Daniel Ribeiro, Silvio E. Perea and João T. Barata

Fig 1D

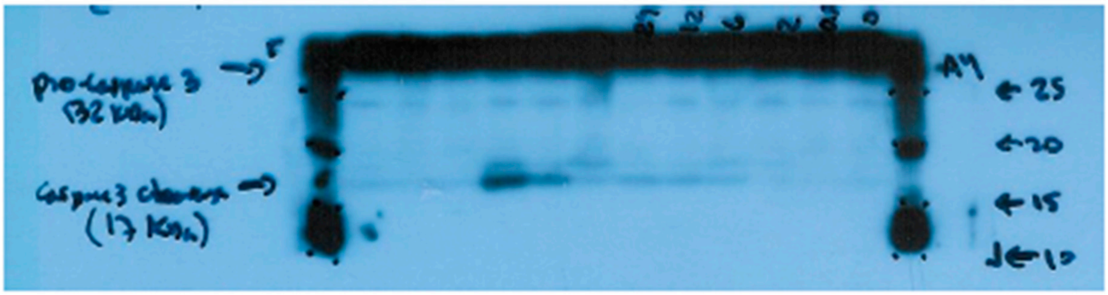

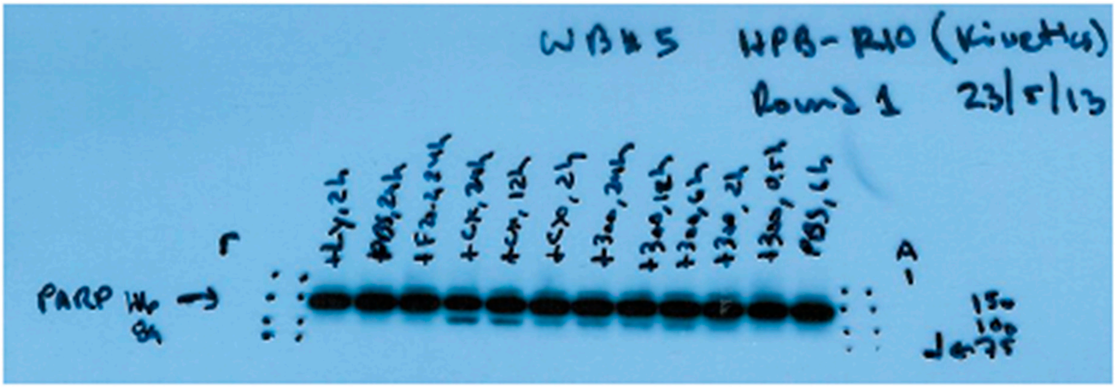

Fig 2A

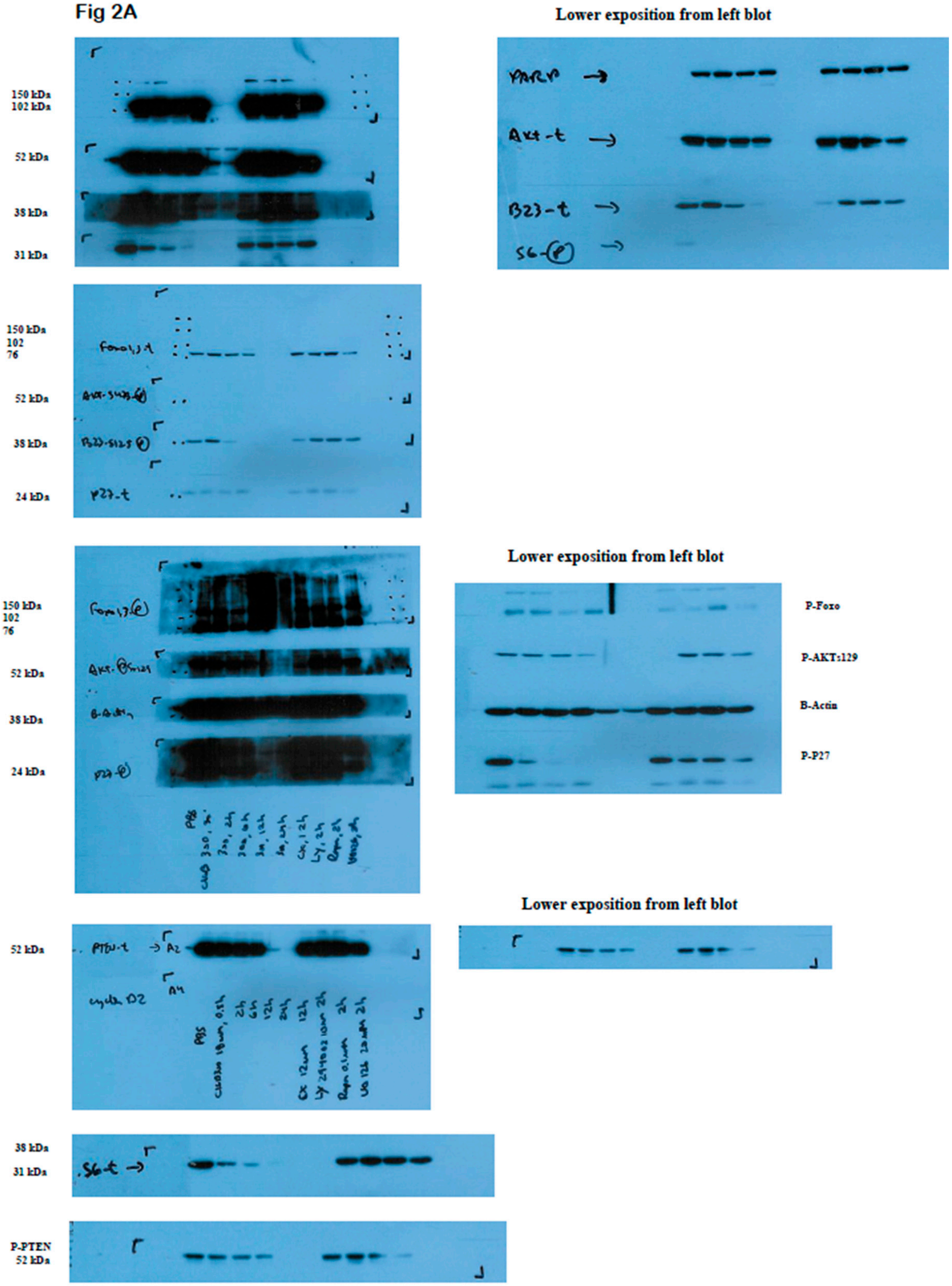
Lower exposition from left blot
150 kDa
102 kDa
52 kDa
38 kDa
31 kDa
PARP
Akt-t
B23-t
150 kDa
102
76
52 kDa
38 kDa
24 kDa
p27-t
Lower exposition from left blot
P-Foxo
P-AKT:129
B-Actin
P-P27
PBS
Lower exposition from left blot
52 kDa
PTEN-t
38 kDa
31 kDa
S6-t
P-PTEN
52 kDa

Fig 2B

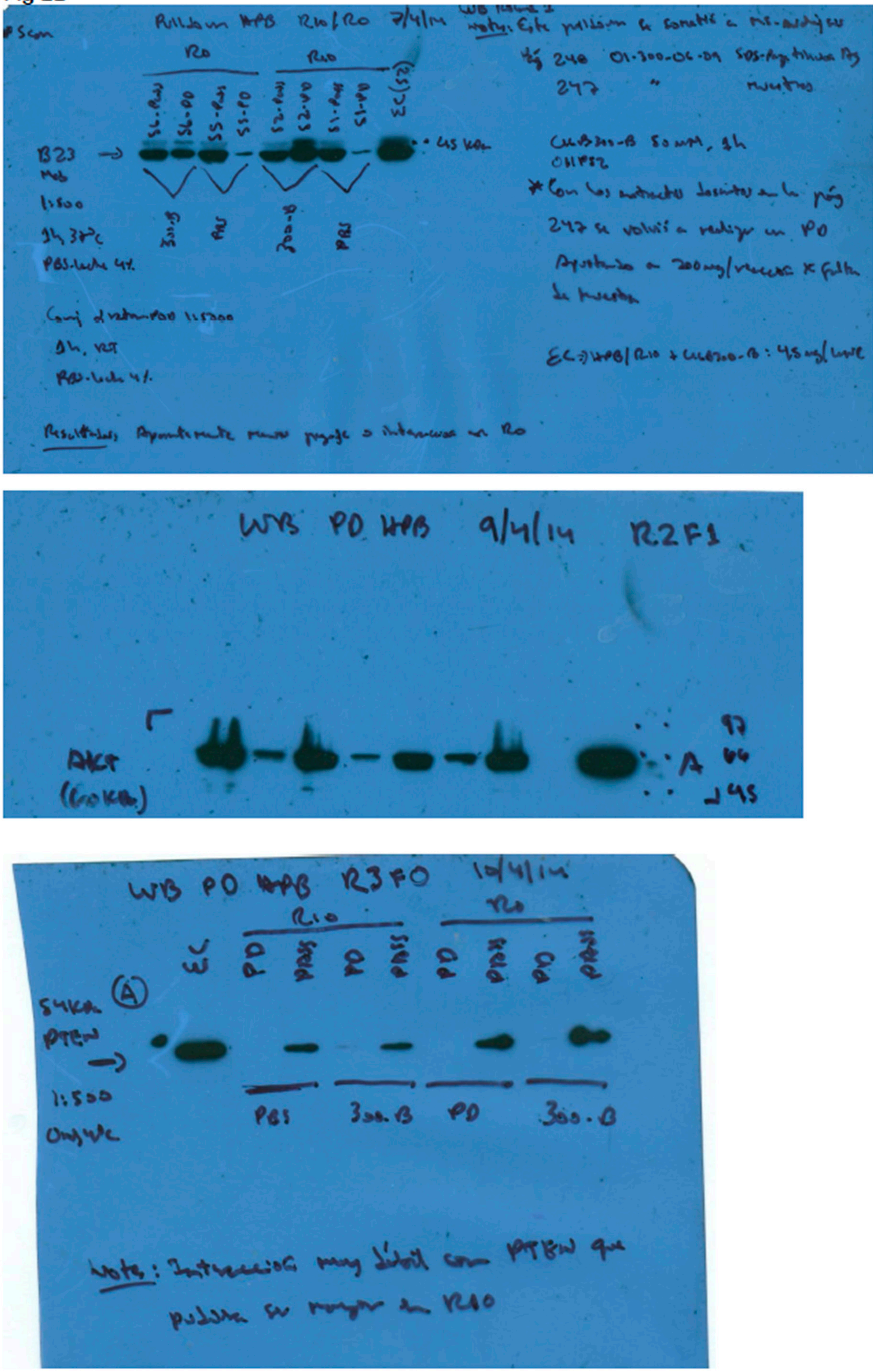
R0
R10
B23
45 KDa
1:500
WB PD HPB 9/4/14 R2F1
AKT
(60KDa)
97
66
45
WB PD HPB R3F0 10/4/14
R10
R0
EC
54KDa
PTEN
1:500
PBS
300-B
PD
300-B

Fig 2C

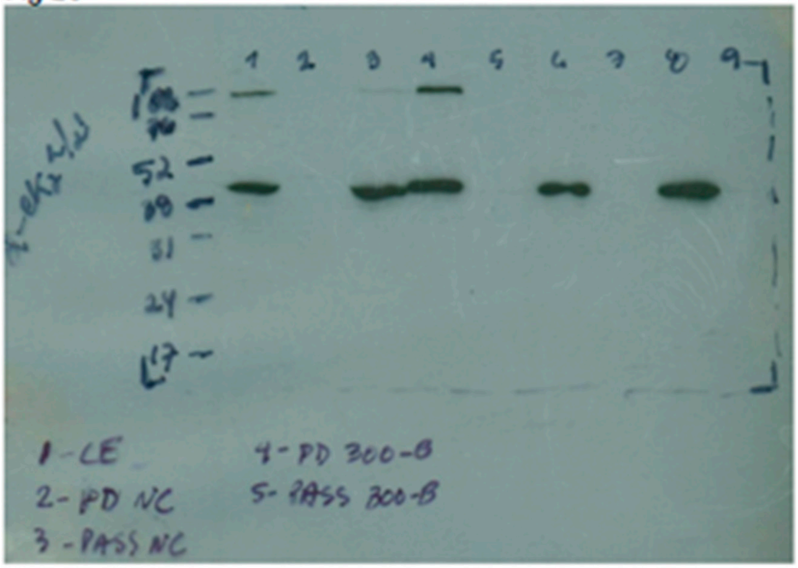

Fig 3B

Unsorted cells

B23/NPM1

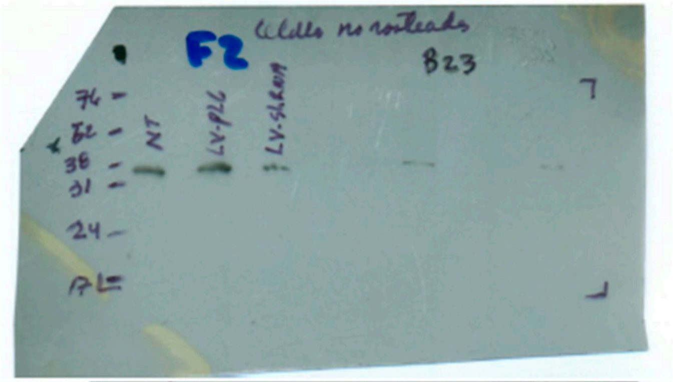

B-Actin

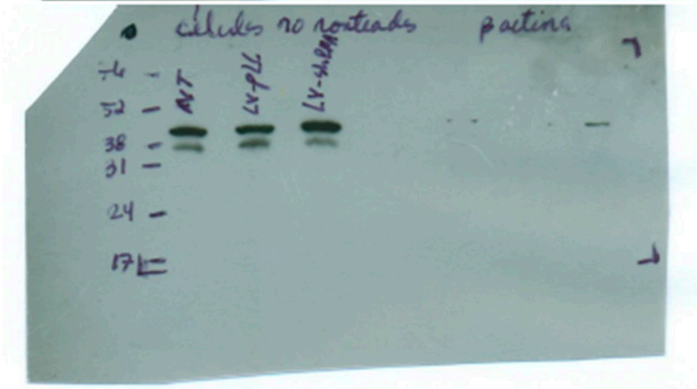

Sorted cells

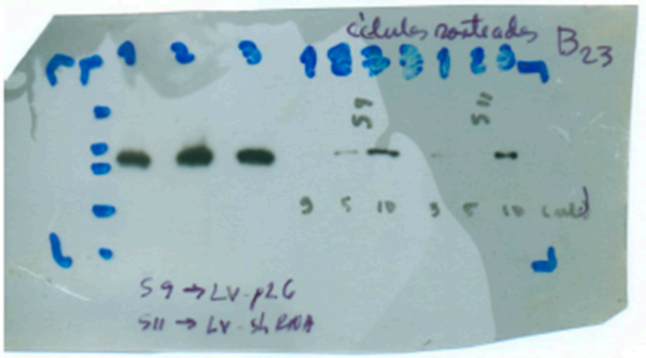

B23/NPM1

Load (µL)

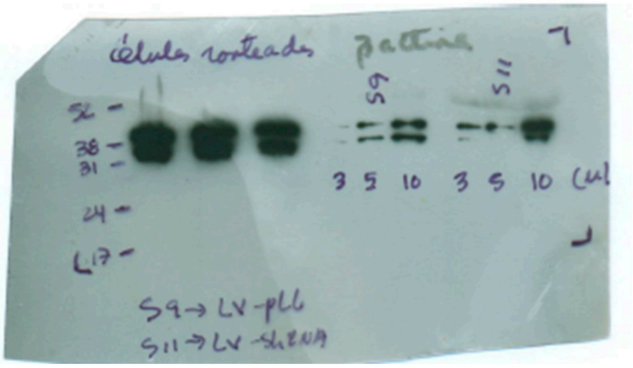

B-Actin

Load (µL)

Fig 4C

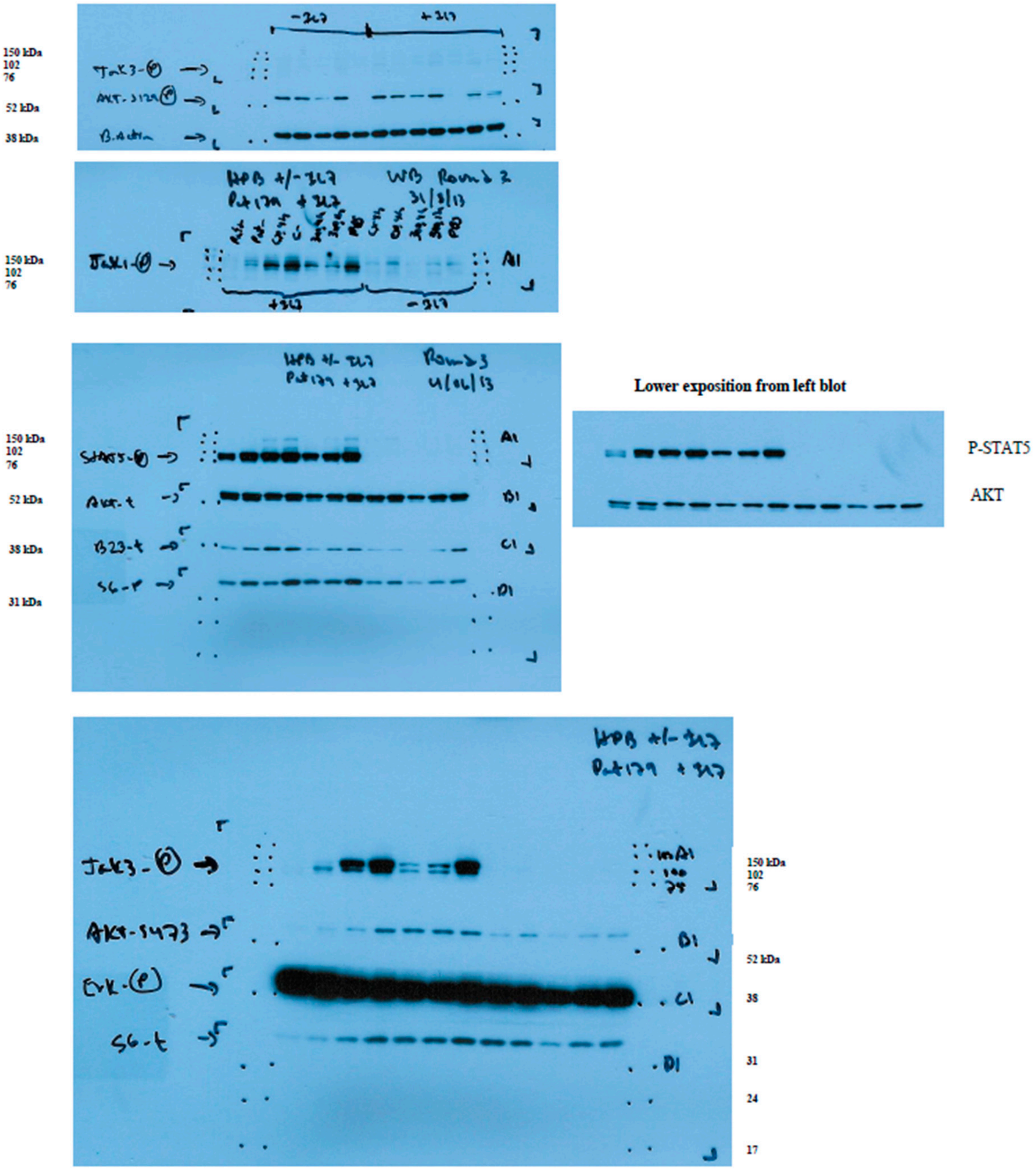
150 kDa
102
76
52 kDa
38 kDa
31 kDa
Lower exposition from left blot
P-STAT5
AKT
24
17

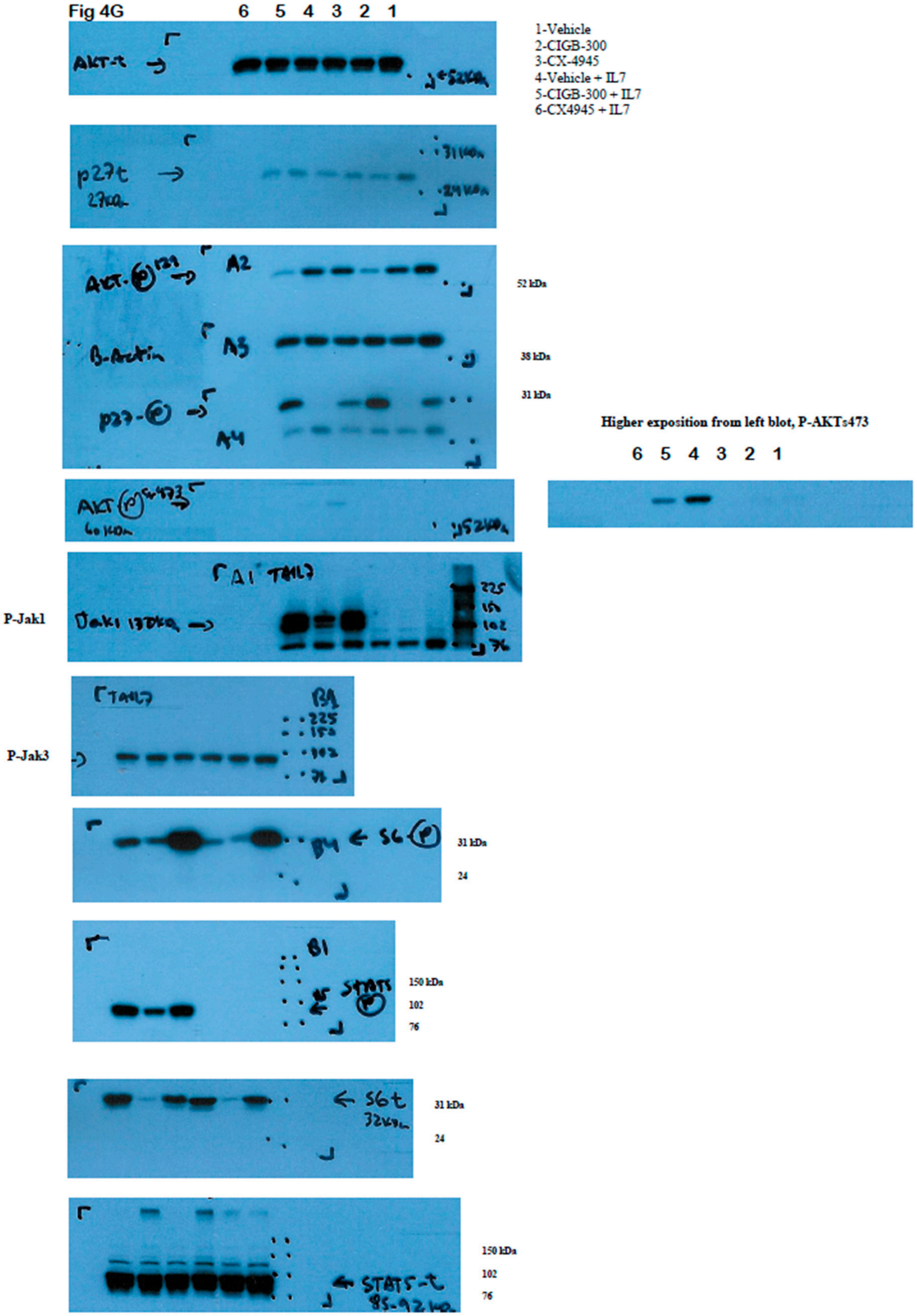
Fig 4G
6 5 4 3 2 1
1-Vehicle
2-CIGB-300
3-CX-4945
4-Vehicle + IL7
5-CIGB-300 + IL7
6-CX4945 + IL7
AKT-t
p27t
52 kDa
38 kDa
31 kDa
B-Actin
Higher exposition from left blot, P-AKTs473
6 5 4 3 2 1
P-Jak1
225
102
76
P-Jak3
31 kDa
24
150 kDa
102
76
S6t
31 kDa
24
150 kDa
102
76
STAT5-t

Fig 6C-Only for patient 179, lanes 1 and 2 here (Pat 179+ means treated with CIGB-300)

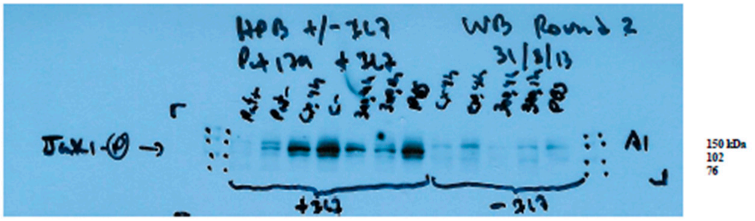

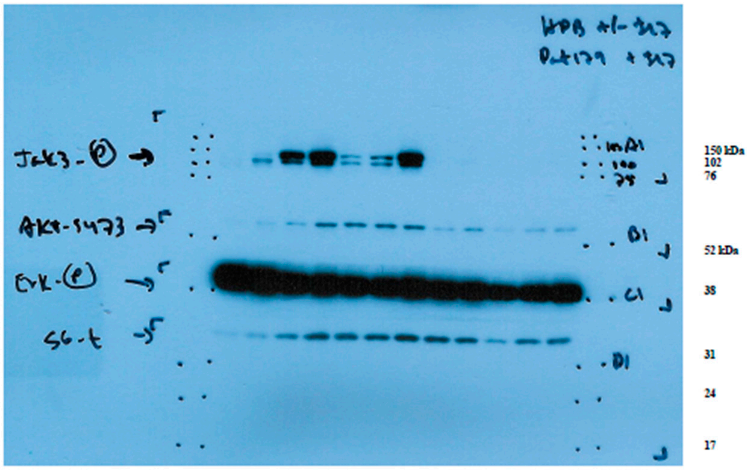

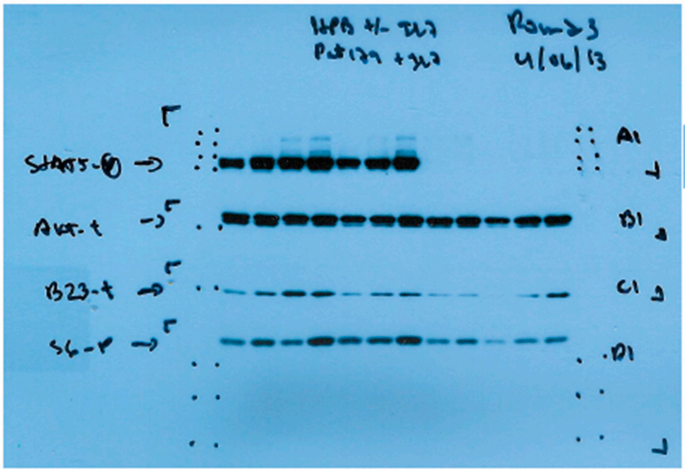

Lower exposition from left blot, P-STAT5

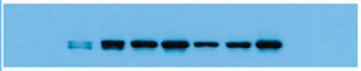

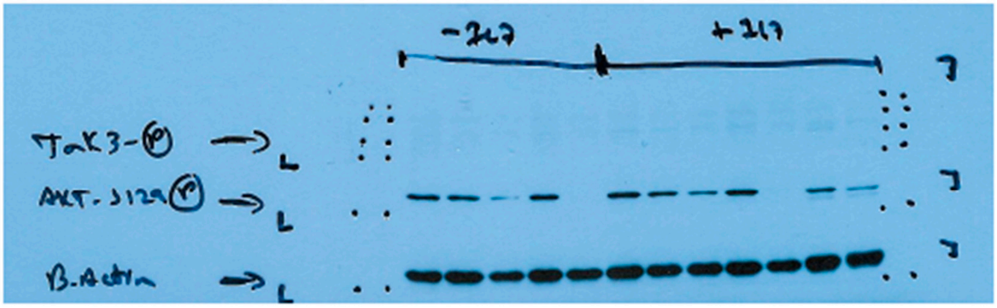

In this last film, patient samples are the final two, in the order: vehicle, then treated with CIGB-300

Figure S1. Detailed information about Figure 1D, 2A-C, 3B, 4C,G, 6C.

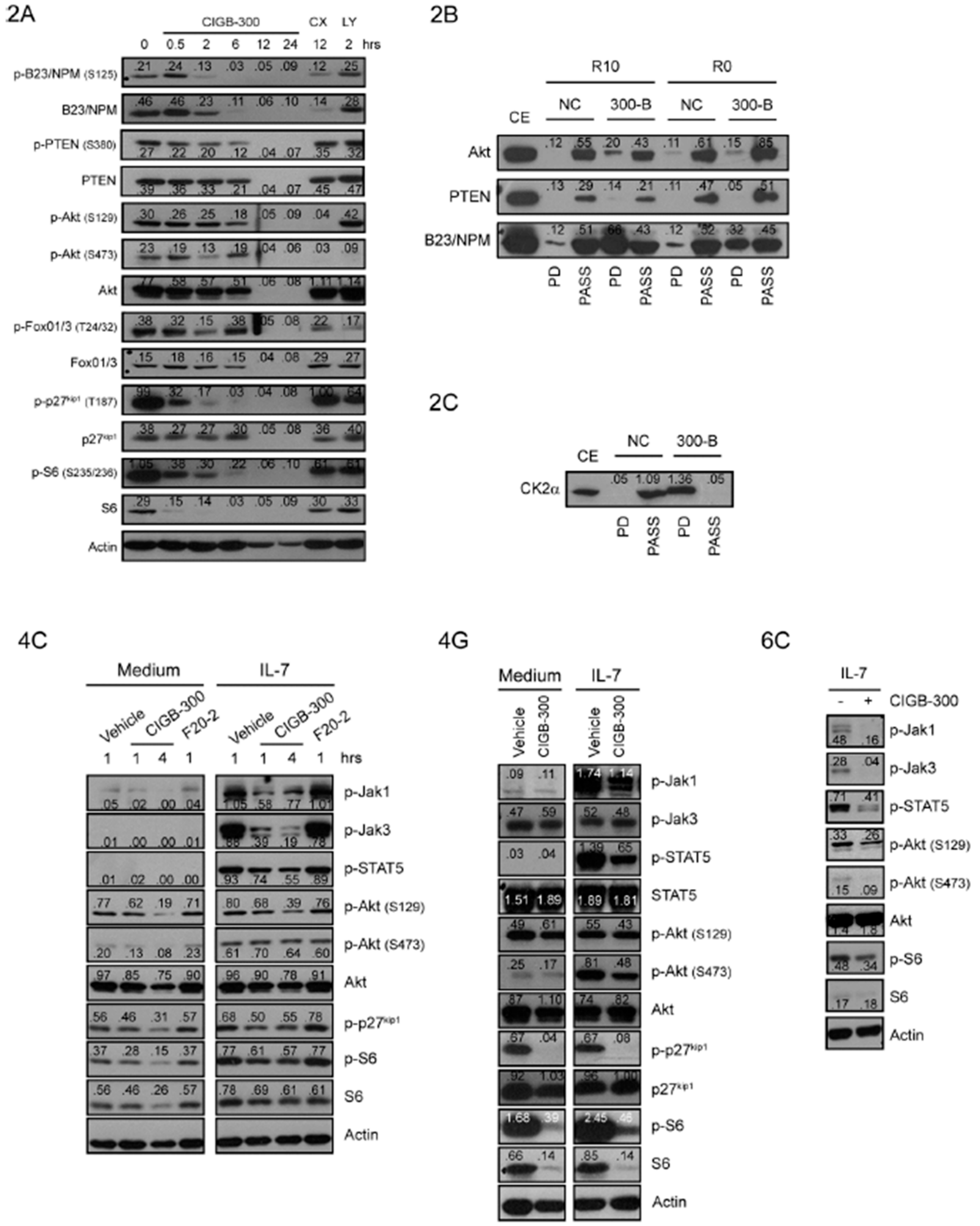

Figure S2. Densiometry analysis values for Figures 2A, 2B, 2C, 4C, 4G and 6C. Densiometry analysis and normalization was performed as indicated in the Methods.

© 2020 by the authors. Licensee MDPI, Basel, Switzerland. This article is an open access article distributed under the terms and conditions of the Creative Commons Attribution (CC BY) license (http://creativecommons.org/licenses/by/4.0/).
